# Systematic evaluation of analytical methods for CSF proteomics

**DOI:** 10.21203/rs.3.rs-7031998/v1

**Published:** 2025-07-15

**Authors:** Aastha Aastha, Leonardo Jose Monteiro Macedo Filho, Michael Woolman, Vladimir Ignatchenko, Alexander Keszei, Gabriela Remite-Berthet, Alireza Mansouri, Thomas Kislinger

**Affiliations:** University of Toronto; Penn State Milton S. Hershey Medical Center; Princess Margaret Cancer Centre, University Health Network; Princess Margaret Cancer Centre, University Health Network; Princess Margaret Cancer Centre, University Health Network; Penn State Milton S. Hershey Medical Center; Penn State Milton S. Hershey Medical Center; University of Toronto

## Abstract

Cerebrospinal fluid (CSF) provides a unique window into brain pathology, yet challenges in unbiased mass-spectrometric (MS) discovery persist due to sample complexity and the need for optimized analytical workflows. Multiple laboratory workflows have been developed for CSF proteomics, each with distinct advantages for specific applications. To interrogate which laboratory workflow is most suitable for this biological matrix, we benchmarked five orthogonal sample-preparation strategies—MStern, Proteograph^™^ nanoparticle enrichment (Seer), *N*-glycopeptide capture (N-Gp), and two extracellular-vesicle (EV) fractions isolated by differential ultracentrifugation (P20- and P150-EV)—in CSF from 19 patients with central nervous system lymphoma. The protocols span a practical spectrum of input volume (6000–50 μL), hands-on time, and reagent cost, enabling informed method selection for translational applications. In total we performed 82 LC-MS/MS experiments and detected over 38,000 unique peptides and more than 3000 proteins across all modalities. Seer achieved the best proteomic depth (~ 17,000 unique peptides) and the tightest detection across samples, followed by P20-EV (~ 9,000), MStern (~ 5,500), P150-EV (~ 5,000), and N-Gp (~ 1,000). None of the methods introduced systematic bias in peptide or protein isoelectric point or hydrophobicity, yet each selectively highlighted distinct biological niches: P20-EVs favoured mitochondrial signatures, N-Gp capture lysosomal and plasma membrane signatures and Seer enhanced nuclear representation. These findings demonstrate that no single protocol suffices for every research question; instead, workflow selection should align with sample-volume constraints, budget and biological question. Our comparative framework empowers investigators to match CSF proteomics strategies to specific neuro-oncological objectives, thereby accelerating the translation of CSF biomarkers into clinically actionable assays.

## Introduction

Cerebrospinal fluid (CSF), continuously produced by the choroid plexus and circulating through the ventricular system and subarachnoid space, provides mechanical protection, metabolic support, and waste removal while maintaining a stable ionic environment crucial for neuronal function (1, 2). Its close interaction with brain tissue and associated pathology makes CSF an invaluable source of biomarkers for neurological disorders, including brain tumors (3, 4).

CSF-based biomarker discovery has gained significant momentum in recent years, with applications ranging from early disease detection to treatment response monitoring and prognostication (5, 6). Two complementary discovery routes dominate current research: (i) targeted assays that quantify predefined molecules with high analytical sensitivity, and (ii) untargeted mass-spectrometry (MS) workflows that chart thousands of proteins, peptides or metabolites in an unbiased fashion. Yet the heterogeneity of CSF composition and the profusion of available enrichment chemistries make it clear that no single pipeline suffices for every clinical question. The growing body of CSF biomarker studies with inconsistent methodologies has impeded reproducibility and clinical translation, underscoring the critical need for standardized protocols (7).

A principal challenge is the fluid’s dynamic range, where concentration differences separate albumin and immunoglobulins from low abundance signalling proteins. High-abundance depletion kits improve depth but can co-deplete interacting analytes and add cost or variability (8). Enrichment strategies that focus on specific sub-proteomes—glycoproteins, or extracellular-vesicle (EV) cargo—offer orthogonal windows into disease biology. Glyco-capture approaches have revealed brain-specific *N*-glycopeptides that would be missed by bulk digests (9), while differential ultracentrifugation of CSF EVs expands measurable protein depth by an order of magnitude and uncovers compartment-specific pathogenic signatures (10).

Despite the development of various approaches to address these analytical challenges, systematic evaluation of CSF proteomic methodologies remains critically limited. This paucity of comprehensive comparative studies hampers the field’s progress, as researchers often select protocols based on convenience or familiarity rather than empirical evidence of superiority for specific biomarker classes. The inherently limited volume of CSF available from clinical samples further necessitates optimized workflows that maximize information yield from minimal input, making method selection particularly consequential for biomarker discovery outcomes in neuro-oncological contexts. To address this knowledge gap, we selected five distinct methods that represent different enrichment philosophies: MStern blotting for standardized bulk proteome processing with proven CSF compatibility using as little as 50 μL sample volumes (11, 12); nanoparticle-based Proteograph^™^ enrichment (Seer) for automated, high-throughput fractionation (seer.bio); *N*-glycoproteomics for targeted capture of brain-derived *N*-glycosylated proteins (13); and two EV fractionation approaches (P20 and P150) for accessing compartmentalized protein signatures from different vesicle populations (14).

In the present study we exploit the relatively large CSF volumes obtained through our multi-agent intraventricular chemotherapy programme to compare the five preparative workflows in CSF samples from 19 central nervous system lymphoma (CNSL) patients. By assessing protein-detection breadth, reproducibility, biophysical bias and subcellular enrichment for each workflow, we delineate the degree to which these methods deliver complementary versus redundant information. The resulting performance map provides practical guidance for selecting and integrating CSF proteomic strategies in neuro-oncology and lays the groundwork for standardised, high-yield protocols that can accelerate biomarker discovery and ultimately improve the clinical management of CNS malignancies.

## Methods

### Sample collection

Three distinct cohorts were recruited for this study to address different experimental objectives. All CSF samples were collected via Ommaya reservoir using standardized procedures at Penn State Hershey Medical Center.

**Cohort 1** (n = 5) was utilized for optimizing EV isolation protocols and determining optimal sample input volumes for ultracentrifugation-based EV enrichment. The median age was 54 years (range: 36–72 years).

**Cohort 2** (n = 11) was utilized for reproducibility studies and biophysical characterization of isolated EVs. All patients in this cohort had central nervous system lymphoma (CNSL). The median age of this cohort was 57 years (range: (41–82 years).

**Cohort 3** (n = 19) served as the primary analytical cohort for comparative evaluation of the five methods across individual patients. This cohort consisted of 19 CNSL patients with a median age of 63 years (range: 25–84 years).

Detailed clinical characteristics for all cohorts are provided in **Supplementary Data 1**.

### Proteomic profiling

#### Sample preparation

##### MStern

Sample processing followed previously established methods (11, 12). In brief, protein concentration in cerebrospinal fluid (CSF) samples was determined using the Pierce BCA assay. For each sample, 50 μL of CSF was processed with the addition of 2 pmol *Saccharomyces cerevisiae* invertase 2 (SUC2) as an internal processing standard. Protein reduction was carried out using 5 mM dithiothreitol for 30 minutes at 60°C, followed by alkylation with 25 mM iodoacetamide for 30 minutes at room temperature in darkness. Protein purification employed a modified MStern protocol (15) using a polyvinylidene fluoride (PVDF) 96-well plate (Millipore) with vacuum suction. The membrane was pre-conditioned with 100 μL of 70% ethanol and washed twice with 100 mM ammonium bicarbonate (ABC) buffer. After protein binding, samples were washed with 100 mM ammonium bicarbonate (pH 8.0) and digested for 4 hours at 37°C with 2 μg MS-grade Trypsin-LysC (Promega) in 50 μL digestion buffer containing 5% acetonitrile, 100 mM ammonium bicarbonate, and 1 mM calcium chloride. Peptides were eluted using 50% acetonitrile and dried by vacuum concentration. Desalting was performed with C18 stage-tips (3M Empore^™^), followed by lyophilization and reconstitution in 0.1% formic acid in LC-MS grade water. Peptide concentrations were measured using a Nanodrop 2000 spectrophotometer (Thermo Scientific). Biognosys iRT peptide standards were added to the reconstituted peptides at a 1:100 dilution as per manufacturer’s guidelines.

##### N-Glycoproteomics

*N*-glycopeptide enrichment was conducted following previously established protocols (16). Lyophilized peptides were reconstituted in coupling buffer (100 mM sodium acetate, 150 mM sodium chloride, pH 5.5) and treated with 10 mM sodium metaperiodate for 30 minutes at room temperature in darkness to oxidize glycans. Excess sodium metaperiodate was eliminated through C18 desalting, followed by lyophilization. Oxidized glycopeptides were then incubated with hydrazide beads overnight under constant rotation. After discarding non-glycosylated peptides, bead-bound *N*-glycopeptides underwent multiple wash cycles with various buffers to remove non-specific peptides. *N*-glycopeptides were subsequently enzymatically de-glycosylated and released from the beads using PNGase F (Roche) in ammonium bicarbonate buffer overnight at 37°C. The resulting de-glycosylated peptides were desalted using C18 stage tips and lyophilized for further analysis. Prior to LC-MS/MS analysis, iRT peptide standards (Biognosys) were spiked into reconstituted peptides at a 1:100 dilution according to manufacturer’s instructions.

#### EV isolation

##### Sample input optimization

CSF-derived EVs were isolated from CSF pools (cohort 1) of increasing volume (1.5 mL, 5 mL, 7 mL and 9 mL). Phosphate-buffered saline (PBS) was used to supplement each sample to a final volume of 11 mL. EVs were isolated by differential ultracentrifugation as previously described (14). Briefly, CSF was centrifuged at 20,000 × g for 30 minutes at 4°C in an Optima XPN-90 ultracentrifuge (Beckman Coulter) equipped with a SW41 Ti swinging bucket rotor to pellet EVs (P20). The P20 pellet was resuspended in 1mL of cold PBS and centrifuged at 18,210 × g for 30 mins. The supernatant from the first centrifugation step was passed through a 0.22 μm filter and centrifuged at 150,000 × g for 2 h at 4°C (SW41TI swinging bucket rotor) in an ultracentrifuge to pellet EVs. The 150,000 × g pellet (P150) was resuspended in high pH buffer (100 mM sodium carbonate buffer, pH 11) and centrifuged again at 150,000 × g for 2 hours at 4°C to pellet P150. The P20 and P150 pellets were resuspended in 100 μL of 50% 2,2,2-trifluoroethanol in PBS. EVs were lysed by freeze-thaw, then incubated at 60°C for 1 hr to extract proteins. Proteins were then reduced with 5 mM of DTT, alkylated with 25 mM of iodoacetamide, and digested overnight at 37°C with a 2 μg Trypsin/Lys-C enzyme mix (Promega). The next day, the enzymatic digest was quenched with 1% formic acid, and samples were desalted with homemade C18 stage tips prior to LC-MS/MS analysis. iRT peptide standards (Biognosys) were spiked into reconstituted peptides at a 1:100 dilution according to manufacturer’s instructions.

##### EV isolation for biophysical characterization and proteomics

Frozen CSF supernatant (6 mL) from cohort 2 and 3 was thawed at 4°C, then diluted to a volume of 11 mL with phosphate-buffered saline (PBS) and processed as described above.

##### EV isolation using Mag-Net protocol

CSF EVs were enriched using Mag-Net protocol that used MagReSyn^®^ strong anion exchange (SAX) beads as previously described (17). Briefly, 300 μL of CSF was mixed with an equal volume of binding buffer (100 mM Bis-Tris Propane pH 6.3, 150 mM sodium chloride) and incubated with pre-equilibrated MagReSyn^®^ SAX beads (1:4 ratio- volume beads to starting CSF volume) (ReSyn Biosciences) for 30 mins. After magnetic separation, the bead-bound extracellular vesicles were washed three times with equilibration buffer (50 mM Bis-Tris Propane pH 6.3, 150 mM sodium chloride) to deplete high-abundance soluble proteins. Vesicle lysis was performed in 300 μL of lysis buffer (50 mM Tris pH 8.5, 1% sodium dodecyl sulfate) with 10 mM tris(2-carboxyethyl) phosphine at 37°C for 60 mins, followed by alkylation with 15 mM iodoacetamide. Proteins were precipitated on-bead by adding acetonitrile (70% final concentration) and washed extensively with 95% acetonitrile and 70% ethanol. On-bead digestion was conducted with trypsin (1:50 ratio) in 100 mM ammonium bicarbonate overnight at 37°C. Peptides were purified using C18 stage tips, dried, and analyzed by LC-MS/MS.

#### Proteograph XT - Seer

The Proteograph XT workflow was applied to 240 μL CSF samples following manufacturer’s protocol (Seer, Inc.). The SP100 automated liquid-handling platform was employed to dispense samples and controls into 96-well format plates. Each CSF sample was incubated with two distinct nanoparticle populations, yielding paired proteomics fractions (NPA and NPB) per specimen. Following nanoparticle-protein corona formation, magnetic immobilization was employed to retain nanoparticle complexes while non-specifically bound material was removed through sequential buffer washes. Captured proteins underwent standard proteomics preparation including reduction, alkylation, and enzymatic digestion using trypsin/LysC, followed by recovery into collection plate. Peptides were dried down in a speed-vac and stored at −80°C until reconstitution. For subsequent analysis, the dried peptides were reconstituted in 25 μL of LC buffer containing 0.1% formic acid (FA) and iRT standard peptides were spiked in at a 1:100 dilution.

##### Mass spectrometry data acquisition and analysis

Peptide samples were introduced onto a 2 cm PepMap Acclaim capture column (Thermo Scientific) via Easy1000 nanoLC system (Thermo Scientific). Chromatographic separation was achieved using a reverse-phase gradient on a 50 cm EasySpray ES903 analytical C18 column (Thermo Scientific) interfaced with a QExactive Orbitrap mass spectrometer (Thermo Scientific). Data acquisition parameters are summarised in **Supplementary Data 2**. Mass spectrometry data were processed with the MaxQuant software (version 2.4.2.0) using a combined database containing the complete UniProt human proteome (v2022_04), yeast invertase (SUC2), and synthetic iRT peptide sequences (18). Search parameters included allowance for up to two missed cleavages, with carbamidomethylation set as a constant modification and methionine oxidation plus N-terminal acetylation as variable modifications. For *N*-glycopeptide enrichment analyses specifically, asparagine deamidation to aspartic acid was additionally set as a variable modification to account for PNGase F-mediated cleavage during elution. A target-decoy approach using reverse sequence databases was employed to maintain false discovery rates below 1% at the site, peptide, and protein identification levels. For all analyses except *N*-glycopeptide enrichment, peptides.txt output files from MaxQuant were processed through an in-house database system for protein grouping. A minimum threshold of two peptides per protein was required for inclusion in downstream analyses. For *N*-glycopeptide analyses, the AspN-AspSites.txt file was utilized, and peptides containing asparagine deamidation modifications within the canonical *N*-glycosylation motif *N*-[!*P*]-*STC* (where *N* = asparagine, [!*P*] = any amino acid except proline, and STC = serine, threonine, or cysteine at position + 2) were identified as *N*-glycopeptides. Only peptides with localization probability scores exceeding 0.8 were retained for subsequent analyses. Quantification of proteins (MStern, Seer, P150- and P20-EV) was achieved by summing the intensities of their constituent peptides, and for *N*-glycoproteins, by summing the intensities across all *N*-glycosites. The processed proteomics data supporting our findings are provided in **Supplementary Data 3**. All downstream analyses employed log_2_-transformed intensity values unless specified otherwise.

##### Nanoparticle tracking analysis

Nanoparticle tracking analysis (NTA) was conducted to assess the concentration and size distribution of P20- and P150-EV fractions. CSF was pooled (6 mL total volume) from 11 patients from cohort 2 (**Supplementary Data 1**). Analysis was performed using a NanoSight NS300 system (Malvern Panalytical) equipped with a 405 nm laser and high sensitivity sCMOS camera. Camera settings were optimized for each fraction: camera level 14, green laser, with slider gain set to 295 for P20-EVs and 245 for P150-EVs. Samples were diluted in 0.22 μm filtered PBS and introduced with a syringe pump at 20 μL/s. The samples were analyzed using NTA software (v3.4 build 3.4.4) at a controlled ambient temperature of 22°C. For each EV fraction, one technical replicate was evaluated. Each replicate consisted of three 30-second video captures, with particle counts maintained between approximately 20–200 particles per field of view for each measurement to ensure optimal tracking accuracy.

Raw data files (“filename-ExperimentSummary.csv”) underwent processing for quantification and statistical analysis. Particle counts for each size bin were normalized using appropriate dilution factors (4 for P150-EV and 5 for P20-EV). Mean values and standard errors were calculated across technical replicates for each experimental group using R (v.4.3.0). Data from both EV fractions were combined based on bin center values to enable comparative analysis. Each data point in the final analysis represents the mean value across all technical measurements per fraction.

##### Negative stain electron microscopy

Negative stain transmission electron microscopy was performed at Princess Margaret Cancer Research Centre for the pooled samples from cohort 2. For sample preparation, 3 microliters of each specimen was placed on carbon-coated grids glow discharged for 10 seconds at 15 mA (PELCO easiGlow) and allowed to absorb for 2 minutes before blotting away bulk solution followed by a single water wash, and two washes with 2% (w/v) uranyl acetate solution. Grids were air dried prior to imaging. Image acquisition was performed on a Talos L120C (ThermoFisher) with a Ceta 4M camera at 57,000x nominal magnification. All images underwent processing with ImageJ software (v. 13.0.6) for optimal visualization.

### Bioinformatic analysis

#### Functional annotation

Each fraction underwent Gene Ontology Biological Process (GO) and subcellular localization annotation for both unique and differentially elevated proteins (DEPs). Fraction-unique proteins were defined as those detected exclusively in a single fraction and absent from all others. For DEP identification, only proteins detected in ≥ 50% of samples per method were included in the analysis. Protein abundance comparisons were conducted using a two-tailed Wilcoxon signed-rank test. P-values were adjusted using the Benjamini-Hochberg (BH) method to control for false discovery rate. Proteins were classified as fraction-elevated when differentially abundant in one fraction compared to all others. For example, a protein was designated as MStern-elevated when it showed significantly higher abundance in MStern compared to each of the other four fractions (Seer, P150-EV, P20-EV, and N-Gp), with fold change > 0 and adjusted p-value < 0.05 for each comparison. The MStern-enriched protein set was therefore defined as the union of MStern-unique and MStern-elevated proteins.

Functional enrichment analysis was performed using compareCluster function from the clusterProfiler R package (v.4.10.1), with the enrichGO method applied to each fraction’s enriched protein set again a background of all CSF detected proteins in cohort 3, i.e. a union of proteins detected in MStern, Seer, P150, P20 and *N*-Glyco fractions. Enrichment analysis was conducted using the GO Biological Process ontology with gene symbols as identifiers and the human genome annotation database (org.Hs.eg.db).

Fraction-enriched proteins were annotated with subcellular localization information derived from the Human Protein Atlas’ Subcellular location data (v.24.0, proteinatlas.org). Eleven main subcellular categories were analyzed: Vesicles, Plasma membrane, Nucleus, Mitochondria, Lysosomes, Golgi apparatus, Endoplasmic reticulum, Cytosol, Cell adhesion, Cytoskeleton, and Cytoplasmic bodies. Several subcellular categories were created by combining related structures based on their biological function and cellular compartmentalization:

- “Nucleus”: Nuclear membrane, Nucleoplasm, Nucleoli, Nuclear speckles, Nuclear bodies, Nucleoli rim, Mitotic chromosome, and Kinetochore
- “Cytoskeleton”: Microtubules, Actin filaments, Intermediate filaments, and Microtubule ends
- “Cell division”: Midbody, Midbody ring, Cleavage furrow, Mitotic spindle, and Cytokinetic bridge
- “Centrosome cilium”: Centrosome, Primary cilium, Basal body, Flagellar centriole, Primary cilium transition zone, Primary cilium tip, and Centriolar satellite
- “Cell adhesion”: Cell Junctions and Focal adhesion sites
- “Cytoplasmic bodies”: Cytoplasmic bodies, Aggresome, and Rods & Rings

For each fraction’s enriched proteins, Fisher’s Exact Test ( fisher.test ) was performed to assess over- or under-representation in each subcellular location. P-values were adjusted for multiple comparisons using the Benjamini-Hochberg method to control the false discovery rate (FDR). The magnitude of enrichment was quantified using log_2_-transformed odds ratios (epitools v.0.5–10.1 , oddsratio.fisher ). A union of proteins detected across all CSF fractions (MStern, Seer, P150, P20, and N-Gp) was used as the background reference set for these analyses.

### Variance analysis

Variance decomposition analysis of the proteomics data was conducted using the variancePartition R package (v.1.14.0, fitExtractVarPartModel function), which employs linear mixed-effects models to partition and quantify protein expression variance attributable to distinct biological and technical factors. The analysis used the formula ~ (1|Method) + (1|PatientID) to assess variance components from experimental method and individual patient effects.

### Brain enriched proteins

Brain-enriched proteins were identified using the Human Protein Atlas database (https://www.proteinatlas.org/humanproteome/brain/human+brain). The curated list of brain-enriched proteins from this resource was used to assess the detection efficiency of brain-specific proteins across different isolation methods.

### Data visualisation

Data visualization was performed using R (v.4.3.0), with figures created through multiple specialized packages: ggplot2 (v3.4.4) for general plots, and ComplexHeatmap (v.2.18.0) for heatmaps, unless otherwise specified.

### Statistical analysis

Statistical comparisons of categorical variables were performed using Fisher’s exact test ( fisher.test ), while continuous variables were analyzed using Wilcoxon rank-sum test ( wilcox.test ). Log_2_ fold changes (log_2_FC) were calculated as the difference between group means, except where indicated otherwise. For multiple testing correction, P-values were adjusted using the Benjamini–Hochberg false discovery rate method for independent tests unless otherwise specified. Correlation analyses were conducted using Spearman’s rank correlation coefficient ( *cor.test* ).

## Results

### Cohort overview and EV input volume optimization

**Figure 1:**
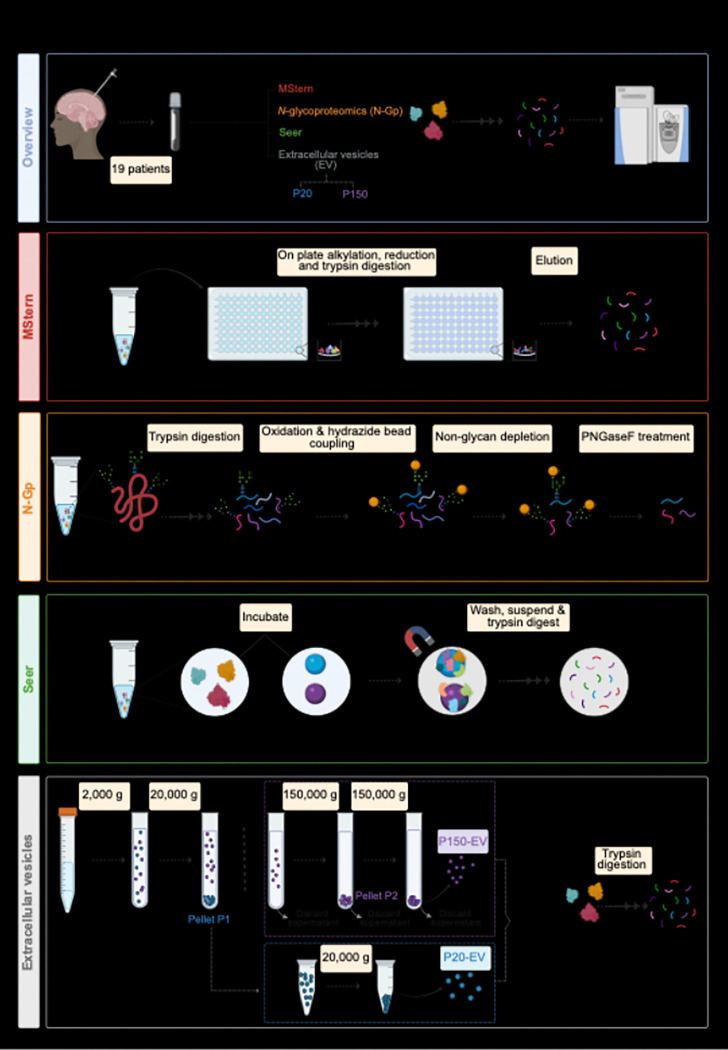
Overview of the experimental workflow Schematic illustrating the sequential steps and enrichment strategies applied to CSF samples—differential ultracentrifugation (P20-EV, P150-EV), MStern, *N-*glycoproteomics (N-Gp) and, Seer Proteograph. Created with Biorender.com.

Here, leveraging CSF from 19 patients (see Methods) with CNS lymphoma, we systematically compared five distinct proteomics methodologies (Fig. 1): MStern (50μL), Seer (250μL), *N*-glycoproteomics (N-Gp, 200μL) and Extracellular Vesicles (EVs)- P150 and P20 (both EV isolation techniques, 6mL).

**Figure 2:**
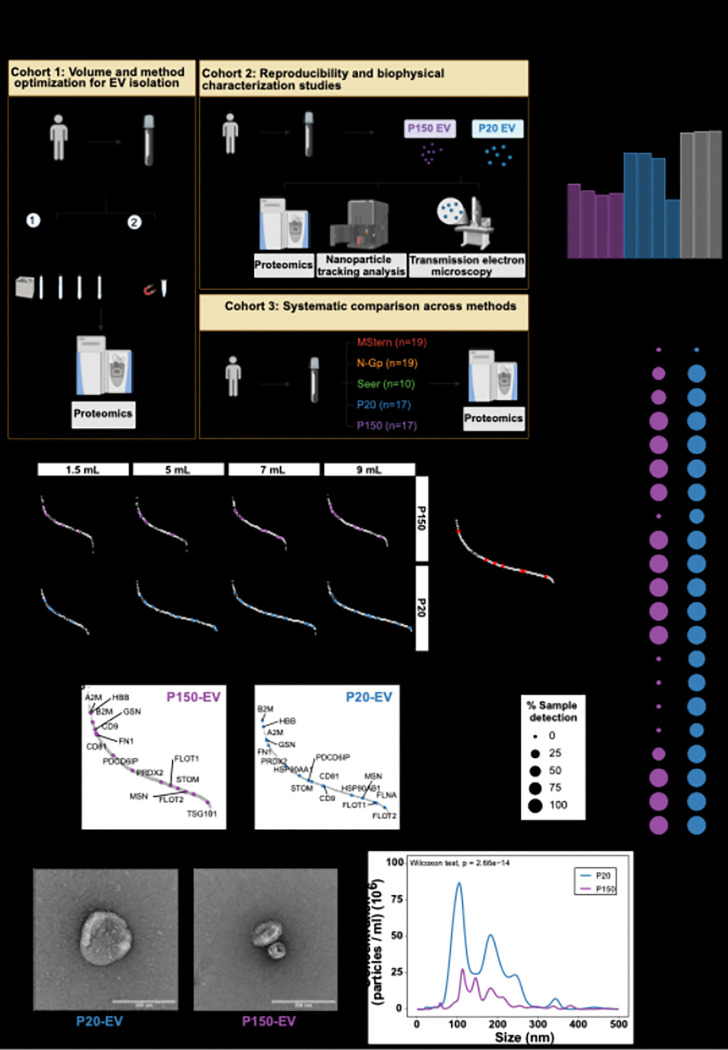
Volume and method optimization for EV isolation from CSF A) Schematic overview of the three patient cohorts and their corresponding experimental workflows. Created with Biorender.com. B) Bar plot summarising the total number of protein groups identified with each isolation strategy: differential ultracentrifugation at 20 000 × g (P20-EV), 150 000 × g (P150-EV) and the Mag-Net protocol. C) Rank-abundance plots for P150-EV and P20-EV fractions from cohort 1 obtained with decreasing CSF input volumes (9, 7, 5 and 1.5 mL), alongside the Mag-Net workflow. Highlighted proteins are known EV markers (20). D) Rank-abundance plots for pooled P150-EV and P20-EV fractions from cohort 2; annotated proteins are established EV markers (20). E) Representative transmission-electron micrographs of P20-EVs and P150-EVs isolated from pooled CSF in cohort 2 (scale bars, 200 nm). F) Nanoparticle-tracking analysis showing particle-size distribution and concentration for P20-EV and P150-EV fractions from cohort 2. G) Dot plot depicting the percentage of cohort 3 samples in which canonical EV markers (20) were detected; dot size encodes detection frequency.

Differential ultracentrifugation is the most widely used method for EV isolation, facilitating size- and density-based fractionation of vesicle populations through sequential centrifugation at varying speeds: 20,000 × g (P20-EV) and 150,000 × g (P150-EV) (19). However, conventional ultracentrifugation-based EV protocols require large volumes (> 10 mL) (14), that exceed typical CSF collections in routine clinical settings. Our team had access to relatively larger volumes of CSF from intraventricular reservoirs implanted as part of our intraventricular chemotherapy program for CNS lymphoma and leptomeningeal metastases. This afforded us the opportunity to conduct volume optimization studies. We first optimized minimum volume requirements using pooled CSF from five patients (cohort 1, Fig. 2A), testing 9-, 7-, 5-, and 1.5-mL starting volumes. Additionally, we evaluated Mag-Net protocol (MagReSyn^®^ SAX beads) (17) as an alternative EV isolation method to assess its performance relative to ultracentrifugation for method selection.

Across the tested volume range of 1.5 to 9 mL, proteomic profiling revealed a notable decline in total protein detection with decreasing input volume, particularly at 1.5 mL, in both P20 (44.5% decrease from 9 mL) and P150 (12.3% decrease from 9 mL) fractions (Fig. 2B). Rank abundance analysis demonstrated that while core EV markers (such as CD9, CD81, PDCD6IP, FLOT1/2) (20) maintained relatively stable rankings across volumes, their detection became increasingly variable at lower input volumes, with several markers falling below detection limits at 1.5 mL (Fig. 2C). While SAX bead-processed samples yielded a higher average protein count (539 proteins) compared to ultracentrifugation fractions at 9 mL (P20-EVs = 449, P150-EVs = 316) (Fig. 2B), the technique showed limitations in selectively enriching specific EV markers such as CD9, PDCD6IP, FLOT1 and FLOT2 (20) (Fig. 2C). Based on these findings, we selected differential ultracentrifugation for all subsequent EV analyses. Volume optimization results showed that while 9 mL provided optimal protein recovery, both 7 mL and 5 mL demonstrated acceptable performance with minimal loss of core EV markers compared to the substantial decline observed at 1.5 mL. We established 6 mL as our standard input volume—representing the maximum volume consistently obtainable from our patient samples while maintaining robust proteomic detection.

Next, to assess reproducibility and methodological consistency, we pooled samples from an independent cohort of 11 CNSL patients (cohort 2) and processed 3 replicates of 6 mL each. CSF EV isolation was highly reproducible, and the two fractions (P150- and P20-EVs) clustered distinctly (**Figure S1A**). We observed consistent detection of known EV markers (Fig. 2D) and a trend toward higher protein detection in P20-EVs (p = 0.07, Wilcoxon test; **Figure S1B**). Biophysical characterization of CSF-derived EVs revealed morphologically similar vesicles in both P20 and P150 fractions (Fig. 2E), though P20-EVs were significantly more abundant (p = 2.66e-14, Wilcoxon test; Fig. 2F). Comparative analysis with urinary EVs using identical isolation protocols (14) revealed similar particle size distributions between CSF and urine (**Figure S1C-D**). While P20-EV concentrations remained comparable between biofluids (**Figure S1C**), P150-EV concentrations were substantially higher in urine compared to CSF (**Figure S1D**).

Finally, having established optimal isolation parameters, we applied our standardized protocol to individual patient samples from cohort 3 (*n* = 17, CNSL diagnoses), the primary cohort for comprehensive proteomic comparisons. EV marker profiling confirmed consistent detection across patient samples (Fig. 2G). In summary, these findings provide a standardized framework for CSF derived EV proteomics that balances analytical sensitivity with practical volume constraints, showing that as little as 6mL input volume can be utilized for detection of EV-specific markers.

### Comparative analysis of proteomic methodologies reveals distinct detection capabilities

**Figure 3:**
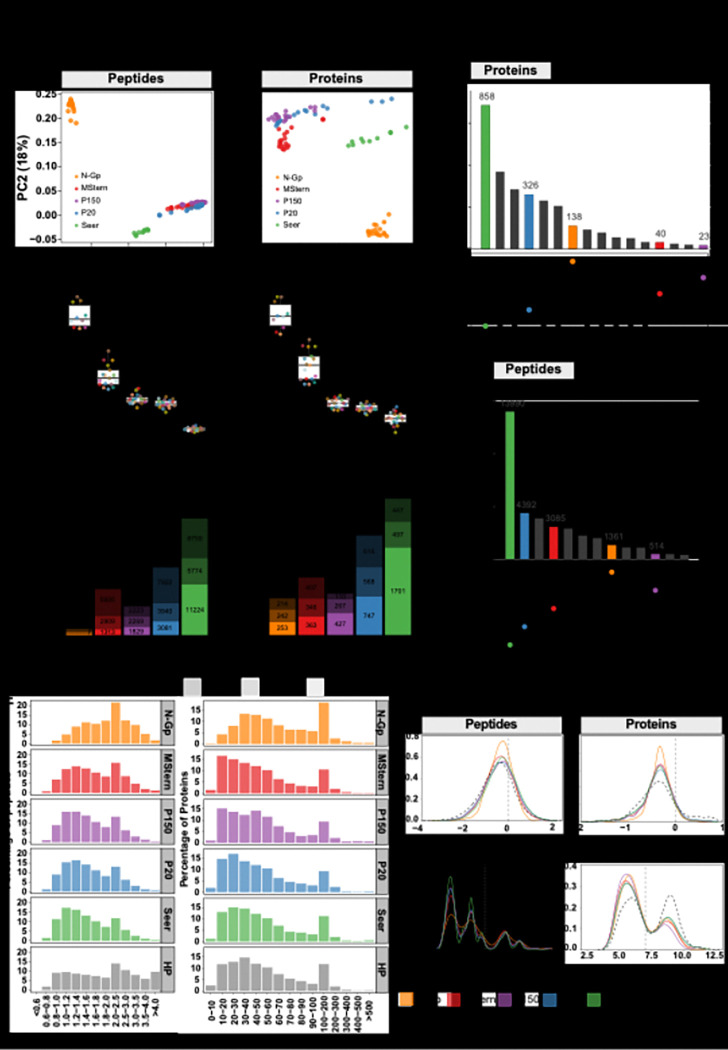
Evaluation of CSF proteome coverage and physicochemical profiles A) Principal-component analysis of log_2_-normalised intensities at the peptide level (left) and protein level (right). Points are coloured by workflow. B) Boxplot showing the median number of unique peptides (left) and protein groups (right) identified per patient sample for each method. Individual dots represent the CSF samples analysed. C) UpSet plots summarising shared and unique identifications across workflows for proteins (top) and peptides (bottom); method-specific (unique) sets are displayed first. D) Stacked bar charts reporting the number of peptides (left) and proteins (right) detected in > 90 % of samples (dark shade), 10–50 % of samples (mid shade) and < 10 % of samples (light shade) for each workflow. E) Histograms of molecular-weight distributions for peptides (left column) and proteins (right column) captured by each method; the in-silico trypsin-digested human proteome is shown in grey for reference. F) Kernel-density plots of GRAVY (hydrophobicity) scores for peptides (left) and proteins (right) across workflows, with the human proteome (dashed line) as reference. G) Kernel-density plots of predicted isoelectric point for peptides (left) and proteins (right), again benchmarked against the reference human proteome (dashed line).

Having established optimal parameters for CSF-EV isolation, we next sought to compare the performance of different proteomic approaches. Principal component analysis revealed method-specific clustering patterns, at both peptide and protein level, likely reflecting the underlying biochemical enrichment strategies (Fig. 3A). To further assess their relative strengths and complementary capabilities, we performed a comprehensive qualitative comparison of protein detection. Substantial differences were noted in detection capacity: Seer proteomics demonstrated the highest average peptide detection capacity, identifying ~ 17,000 peptides, followed by P20-EVs (~ 8,000 peptides), while MStern, P150-EVs, and N-Gp demonstrated progressively lower peptide yields ranging from 5,000 to 1,000 peptides (Fig. 3B). Similar detection patterns were observed at the protein level, with Seer maintaining the highest average protein detection (~ 2,100 proteins), P20-EVs detecting ~ 1,300 proteins, and the remaining methods identifying 500–700 proteins each (Fig. 3B). However, this trend diverged at the protein intensity level, where MStern exhibited the highest median abundance per sample, which may partially explain its limited detection capacity (**Figure S2A**). Consistent with its superior peptide detection, Seer also demonstrated the highest median peptides per protein (> 5) (**Figure S2B**). The predominantly single-peptide representation in N-Gp aligns with the inherently limited glycosylation sites within proteins, that are also detectable following tryptic digestion (**Figure S2B**).

We next analyzed the overlap and uniqueness of peptide and protein identifications across platforms (Fig. 3C). At the peptide level, Seer achieved the highest detection with 13,990 unique peptides, substantially exceeding P20-EVs (4,392), MStern (3,085), N-Gp (1,361) and P150-EVs (514). There were minimal shared identifications across all five methods, with the majority being method specific. Notably, N-Gp showed particularly limited commonality with other approaches, likely because *N*-glycopeptides are typically missed in conventional bulk digestion workflows. In contrast, protein-level analysis demonstrated substantially greater inter-method concordance, with a core set of proteins consistently detected across multiple platforms while each method contributed unique protein identifications. This convergence likely reflects the mapping of multiple distinct peptides to the same protein, potentially enabling shared protein identifications despite largely non-overlapping peptide populations. This may also be influenced by analyzing the same disease context, where common pathological proteins are detected across different enrichment strategies. Next, to assess method reproducibility, we evaluated peptide detection consistency across individual samples (Fig. 3D). Seer demonstrated superior reproducibility with the majority of peptides (11,224 peptides) detected in > 90% of samples, while N-Gp showed the highest variability with substantial peptides detected in only 10–50% of samples, likely reflecting biological heterogeneity in glycosylation patterns and complexity of the enrichment protocol. MStern, P20-EVs, and P150-EVs exhibited intermediate consistency. At the protein level, detection consistency improved across all methods, with Seer maintaining the highest reproducibility and total protein identifications (Fig. 3D).

### Evaluation of physicochemical properties

To evaluate potential physicochemical selection biases, we compared the molecular-weight, hydrophobicity (GRAVY score) and isoelectric-point (pI) profiles of the peptides and proteins detected by each workflow with those expected from an in-silico tryptic digest of the human reference proteome (Fig. 3E–G). The five fractions were broadly concordant with the theoretical baseline; the only pronounced deviation was observed for the N-Gp protocol. At the peptide level, N-Gp was enriched for larger species, with a modal bin of 2.0–2.5 kDa, and this translated into a right-shift in the molecular-weight distribution of the corresponding proteins (Fig. 3E). N-Gp also exhibited a modest, but reproducible, skew towards more hydrophobic sequences, evidenced by a shift to higher GRAVY scores relative to both the in-silico digest and the other enrichment methods (Fig. 3F). The remaining workflows overlapped almost completely with the theoretical proteome for both parameters, indicating minimal bias. The bias of N-Gp towards heavier and slightly more hydrophobic peptides likely reflects the structural context of *N*-linked glycosylation sites: (i) bulky glycans sterically shield neighbouring Lys/Arg residues, causing missed tryptic cleavages and consequently longer peptide backbones; and (ii) glycoproteins are typically secreted or membrane-associated, so the sequon-containing regions they contribute tend to be flanked by hydrophobic stretches. Together these factors enrich the capture pool for higher-mass, more hydrophobic glycopeptides.

Notably, pI profiles revealed a systematic gap at the basic end of the spectrum: the theoretical digest shows a clear second mode above pI 9, yet this peak is virtually absent from all experimental datasets (Fig. 3G). Highly basic peptides elute very early from conventional reversed-phase columns (21, 22) and ionize less efficiently under the acidic conditions (23) used for nano-LC-MS/MS, explaining their universal under-representation regardless of enrichment strategy. Collectively, these data show that all workflows provide largely unbiased physicochemical coverage of the CSF proteome, with the expected selectivity of N-Gp towards longer, modestly more hydrophobic glycopeptides.

### Clinical utility and functional enrichment

**Figure 4:**
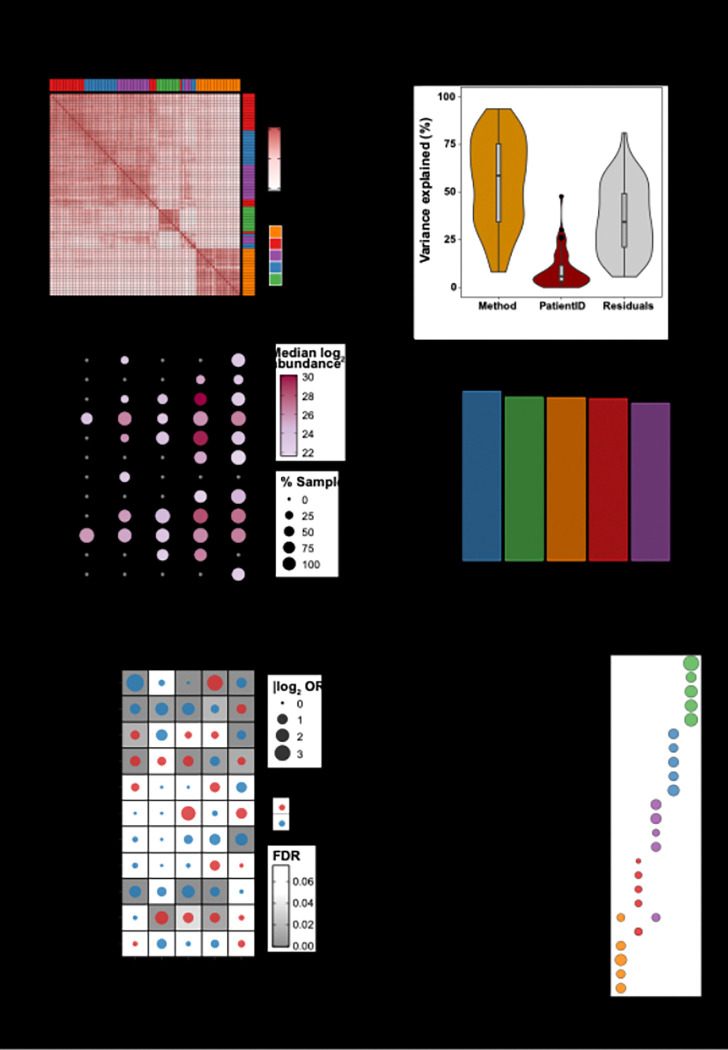
Clinical utility and functional enrichment A) Clustered heatmap of pair-wise Spearman correlation coefficients for all patient–workflow combinations; column and row colours indicate the enrichment method. B) Violin plots (with inset box plots) showing the percentage of total protein-level variance explained by workflow (“Method”), by inter-individual differences (“Patient ID”) and by residual noise, as determined with a linear mixed-effects model. C) Dot plot of brain-enriched proteins (consolidated from the Human Protein Atlas): colour encodes median log_2_ abundance and dot size the percentage of samples in which each protein was detected for the indicated workflow. D) Bar graph depicting percentage of brain detected (24) proteins within each workflow’s CSF proteome. E) Dot plot of gene-set over-representation analysis for selected sub-cellular compartments (odds-ratio, bubble size; FDR, grey scale). Red bubbles denote over-representation, blue bubbles under-representation, based on method-specific differentially expressed proteins (*see* Supplementary Data 4). F) Dot plot of selected GO Biological-Process enrichment terms of the same method-specific protein sets: dot size reflects the number of annotated genes and colour the contributing workflow cluster (*see* Supplementary Data 5).

To evaluate method concordance and variance contributions, we performed correlation analysis and linear mixed modeling. Notably, high inter-method sample-wise and protein-wise correlations were observed among the broad-coverage workflows—MStern, P150, P20 and Seer (r = 0.69–0.81)—whereas N-Gp showed markedly lower agreement with the others (r = 0.52–0.58), consistent with its targeted selectivity (Fig. 4A; **Figure S3A**). Correspondingly, variance decomposition using linear mixed models revealed that methodological choice accounted for over 60% of total variance in protein detection (Fig. 4B). Hierarchical clustering of binary detection calls similarly produced discrete method-specific protein blocks (**Figure S3B**). Collectively, these analyses demonstrate that while a substantial core proteome is shared across approaches, the choice of enrichment workflow represents the dominant source of variation in CSF proteomic characterization.

Beyond methodological performance metrics, we investigated the clinical utility of each approach through analysis of brain-enriched proteins (Fig. 4C). Seer and P20-EVs demonstrated superior detection of brain-enriched proteins (10/12 and 9/12 proteins, respectively), while MStern and P150-EVs showed moderate coverage (7/12 and 6/12). N-Gp exhibited the lowest detection (2/12), which can be attributed to two factors: several brain-enriched proteins lack *N*-glycosylation sites (SYP, MBP, PLP1, GFAP, HCRT, AIF1, and TAFA1), while others that are *N*-glycosylated (ITGAM, HAPLN2, AQP4) were likely detected through their non-glycosylated peptides in other methods but fell below detection limits when relying solely on glycopeptide enrichment. This suggests either low glycosylation occupancy or insufficient enrichment efficiency for these specific glycoproteins. Remarkably, when examining the broader proteome context, all methods demonstrated consistently high detection rates for brain-detected proteins (24) as a percentage of their total identified proteome (Fig. 4D, P20: 84.13%, Seer: 81.21%, N-Gp: 81.00%, MStern: 80.47%, P150: 78.22%). This striking consistency across methodologically distinct approaches likely reflects the inherent brain-centric nature of CSF as a biofluid, where brain-derived proteins constitute the predominant proteomic signature regardless of the analytical strategy employed. We also examined the top 10 most abundant CSF proteins (8) and CNSL-enriched markers identified from our previous studies (**Figure S3C**). CSF-abundant proteins showed consistent detection across most methods (7–10 proteins detected), while CNSL-enriched markers exhibited more variable detection patterns, with Seer and P20-EVs demonstrating superior coverage compared to other approaches.

Intriguingly, each methodology exhibited distinct subcellular signatures that directly reflect their underlying enrichment mechanisms (Fig. 4E–F). P20-EVs demonstrated significant mitochondrial protein enrichment with corresponding enrichment for respiratory chain assembly pathways, while N-Gp preferentially detected plasma membrane, lysosomal, and Golgi proteins, enriching for receptor signaling processes consistent with cell surface glycoprotein targeting. Seer exhibited enhanced nuclear protein representation and RNA processing pathways, whereas P150-EVs and MStern captured vesicular and cytoplasmic proteins with predominantly immune-related pathway enrichment including complement activation (**Supplementary Data 4 & 5**).

## Discussion

This study provides the first systematic, within-cohort comparison of five mechanistically distinct CSF proteomics workflows—MStern, Proteograph^™^ nanoparticle enrichment, N-glycopeptide capture (N-Gp) and two differential-ultracentrifugation fractions (P20-EV and P150-EV). The use of CSF collected from Ommaya reservoirs enhances the clinical relevance of our findings, as it provides direct access to the brain microenvironment with minimal peripheral blood contamination, making it particularly well-suited for neurological biomarker discovery studies (25). By standardizing all downstream LC-MS settings, we ensured that performance differences reflected sample-preparation chemistry rather than acquisition parameters. This comprehensive approach addresses a critical gap in the field, where previous studies have typically evaluated individual methods in isolation, making direct performance comparisons challenging.

Unsupervised PCA revealed that the choice of workflow, rather than inter-patient variability, was the dominant source of variance in the dataset. This finding underscores the profound impact of sample preparation methodology on proteomic outcomes and highlights the importance of method selection in CSF biomarker studies. Combining complementary enrichment chemistries, our integrated workflow identified > 38,000 unique peptides representing ~ 3,000 protein groups, providing a richly detailed CSF proteome for downstream discovery and biological insight.

Each workflow exposed a unique slice of CSF biology, revealing distinct advantages for specific applications. Seer’s two physicochemically distinct beads yielded the broadest coverage and preferentially captured nuclear and chromatin-associated proteins, likely reflecting the technology’s ability to reduce dynamic range compression and enable detection of low-abundance peptides that are typically masked by abundant plasma proteins in CSF. However, the high per-sample cost (>$200/sample – not counting the required liquid handling robot for sample preparation) could present barriers to widespread implementation, particularly for larger cohort studies, making this approach optimal for targeted validation studies or applications where detection of low-abundance, biologically relevant proteins is paramount.

Differential centrifugation yielded two CSF pellets- P20-EVs (20,000g) and P150-EVs (150,000g). Compared to urine—a readily available, extensively profiled biofluid that yields abundant P150-EVs with the same ultracentrifugation workflow (14, 26)—CSF contains substantially fewer P150-EVs, likely reflecting an enrichment for distinct vesicle populations such as synaptic vesicles in CSF. The P20-EV fraction was enriched for mitochondrial pathways, representing a novel observation in CSF-derived extracellular vesicles. While microvesicles are typically isolated at 10,000 to 20,000g (27), the specific enrichment of mitochondrial proteins in this fraction has not been previously reported in CSF. This finding suggests that the P20 fraction captures organelle-derived extracellular vesicles that may reflect cellular stress responses and metabolic dysfunction (14, 28, 29) within the central nervous system, providing new insights into neuroenergetic mechanisms relevant to neurological diseases. In contrast, the P150-EV fraction showed limited protein detection, potentially due to the lower abundance of smaller extracellular vesicles in CSF compared to other biological fluids or methodological challenges in isolating this specific vesicle population from CSF. Both EV methodologies require substantial sample volumes (minimum 6mL), which may be prohibitive in clinical contexts where CSF availability is limited, and their labor-intensive protocols restrict sample throughput, making them most valuable for mechanistic studies focused on understanding vesicle-mediated communication in the CNS.

N-Gp methodology recovered > 1,000 unique glycopeptides, many mapping to lysosomal and receptor-signaling pathways that are typically missed in bulk protein digests yet heavily implicated in CNS pathology (30). This glycoprotein-centric perspective is especially pertinent to neurology, where aberrant protein glycosylation has been linked to the pathogenesis of neurological diseases such as Alzheimer’s disease, amyotrophic lateral sclerosis (ALS), and glioma (31–33). While sample volume requirements are modest (200 μL), the technique is time-intensive, requiring approximately four days to process 20 samples, though adoption of AutoTip-based robotic hydrazide protocol (34) could significantly reduce processing time and enable higher throughput workflows. This approach is particularly valuable for investigations focusing on biomarker discovery and therapeutic research because it enriches disease-relevant glycoproteins—the very class that represents more than two-thirds of FDA-approved biologics (35).

MStern, while not an enrichment method per se, provided balanced proteome coverage at minimal cost and hands-on time, offering high-throughput capabilities (96 samples per plate within 3–4 hours) and cost-effectiveness (15) that make it particularly well-suited for initial discovery phases involving larger cohorts and limited sample volumes. However, its limitations in detecting low-abundance proteins due to overrepresentation of high-abundance plasma proteins should be acknowledged. The integration of data-independent acquisition (DIA) methods significantly improves MStern performance by enhancing quantitative reproducibility and enabling more sensitive detection of low-abundance species through comprehensive spectral libraries, making it particularly attractive for population-scale discovery screens, as demonstrated in high-throughput plasma (36) and urine workflows (37, 38).

Collectively, these findings challenge the notion of a universal “best” protocol for CSF biomarker discovery. Instead, our results support a context-dependent approach where method selection aligns with research objectives, sample constraints, and available resources (Table 1). For comprehensive biomarker discovery programs, a tiered approach may be optimal: initial screening with cost-effective, high-throughput methods like MStern-DIA, followed by targeted validation using more specialized techniques like Seer or N-Gp for specific protein classes of interest. Method selection should be informed by careful consideration of time requirements, resource availability, equipment needs, cost considerations, sample volume limitations, and the biological relevance of target proteins. Future developments in automation, cost reduction, and method optimization may alter this landscape, and the integration of artificial intelligence and machine learning approaches for method selection and data integration across platforms represents a promising avenue for maximizing the complementary strengths of different proteomics workflows while minimizing their individual limitations.

## Figures and Tables

**Table 1:** Comparative analysis of five CSF proteomics sample preparation methods. Methods are compared across key performance metrics including sample requirements, processing characteristics, detection capabilities, and practical implementation considerations. Cost categories: **Low** (<$10/sample), **Medium** ($10–200/sample), **High** (>$200/sample). Equipment complexity reflects specialized instrumentation requirements and technical expertise needed (**High:** Uses standard lab equipment, minimal specialized instrumentation; **Medium:** Requires some specialized equipment but commonly available; **Low:** Requires highly specialized, expensive instrumentation or complex setups). Clinical feasibility considers sample volume requirements, processing complexity, and routine implementation potential.

Method	Sample Volume (μL)	Processing Time	Throughput (Samples/day)	Relative Cost	Protein Detection (count/sample)	Equipment Complexity	Clinical Feasibility
**MStern**	50	4–5 hours	At least 96	Low	~ 700	Low	High
**Seer**	250	4–6 hours	At least 24–48	High	~ 2000	High	Medium
**N-Gp**	200	4 days	20	Medium	~ 400	Low	Medium
**P150-EV**	6000	6–8 hours	6–12	Medium	~ 500	High	Low
**P20-EV**	6000	6–8 hours	6–12	Medium	~ 1300	High	Low

## Data Availability

Proteomic data generated in this study have been deposited to MassIVE (MSV000098387). The corresponding FTP link is ftp://MSV000098387@massive-ftp.ucsd.edu and the password is XY37D12W.
